# Agnoprotein of polyomavirus BK interacts with proliferating cell nuclear antigen and inhibits DNA replication

**DOI:** 10.1186/s12985-014-0220-1

**Published:** 2015-02-01

**Authors:** Nancy Gerits, Mona Johannessen, Conny Tümmler, Mari Walquist, Sergiy Kostenko, Igor Snapkov, Barbara van Loon, Elena Ferrari, Ulrich Hübscher, Ugo Moens

**Affiliations:** UiT - The Arctic University of Norway, Faculty of Health Sciences, Department of Medical Biology, Molecular Inflammation Research Group, Tromsø, NO-9037 Norway; Faculty of Health Sciences, Department of Medical Biology, Host Microbe Interaction Research Group, Tromsø, NO-9037 Norway; Institute of Veterinary Biochemistry and Molecular Biology, University of Zürich-Irchel, Winterthurerstrasse 190, Zürich, 8057 Switzerland; Present address: UiT - The Arctic University of Norway, Faculty of Biosciences, Fisheries and Economics, Norwegian College of Fishery Sciences, Group of Seafood Science, Tromsø, NO-9037 Norway; Present address: University of Bergen, Department of Molecular Biology, Molecular Bioenergetics and Signalling, Bergen, NO-5020 Norway

**Keywords:** Agnoprotein, Cell proliferation, DNA polymerase, DNA replication, PCNA, Polyomaviruses

## Abstract

**Background:**

The human polyomavirus BK expresses a 66 amino-acid peptide referred to as agnoprotein. Though mutants lacking agnoprotein are severely reduced in producing infectious virions, the exact function of this peptide remains incompletely understood. To elucidate the function of agnoprotein, we searched for novel cellular interaction partners.

**Methods:**

Yeast-two hybrid assay was performed with agnoprotein as bait against human kidney and thymus libraries. The interaction between agnoprotein and putative partners was further examined by GST pull down, co-immunoprecipitation, and fluorescence resonance energy transfer studies. Biochemical and biological studies were performed to examine the functional implication of the interaction of agnoprotein with cellular target proteins.

**Results:**

Proliferating cell nuclear antigen (PCNA), which acts as a processivity factor for DNA polymerase δ, was identified as an interaction partner. The interaction between agnoprotein and PCNA is direct and occurs also in human cells. Agnoprotein exerts an inhibitory effect on PCNA-dependent DNA synthesis *in vitro* and reduces cell proliferation when ectopically expressed. Overexpression of PCNA restores agnoprotein-mediated inhibition of cell proliferation.

**Conclusion:**

Our data suggest that PCNA is a genuine interaction partner of agnoprotein and the inhibitory effect on PCNA-dependent DNA synthesis by the agnoprotein may play a role in switching off (viral) DNA replication late in the viral replication cycle when assembly of replicated genomes and synthesized viral capsid proteins occurs.

**Electronic supplementary material:**

The online version of this article (doi:10.1186/s12985-014-0220-1) contains supplementary material, which is available to authorized users.

## Background

Polyomaviruses are naked viruses with a circular double-stranded DNA genome of approximately 5,000 base-pairs. All polyomaviruses isolated so far seem to encode the regulatory proteins large T-antigen and small t-antigen and at least two capsid proteins VP1 and VP2 [1-3]. Some PyVs encode a third capsid protein, VP3 [4]. The human PyVs BK (BKPyV) and JC virus (JCPyV), and the monkey PyV simian vacuolating virus 40 (SV40) encode an additional regulatory protein referred to as agnoprotein [3,5]. Agnoprotein is highly conserved, especially the first 2/3 part of the peptide and is characterized by a hydrophobic central region [6-10]. None of the recently characterized human PyVs (KIPyV, WUPyV, Merkel cell PyV, HPyV 6, HPyV7, trichodysplasia spinulosa-associated polyomavirus, HPyV9, HPyV10, STLPyV and HPyV12) seem to express agnoprotein [11-19]. An open reading frame encoding a putative protein with sequence homology to agnoprotein is also present in the genomes of simian virus 12 (*Papio ursinus*), the white-fronted capuchin polyomavirus (*Cebus albifrons*), yellow baboon polyomavirus 2 (*Papio cynecephalus*), and vervet monkey polyomavirus 2 (*Chlorocebus pygerythrus*), [20-22]. However, the existence of agnoprotein encoded by these non-human primate PyVs remains to be proven.

The exact function of agnoprotein remains unsolved, but studies with SV40 and JCPyV have demonstrated that it is involved in viral DNA replication [23], nuclear egress [24], transcriptional and post-transcriptional processes [25,26], maturation [27,28], protein stability and localization [29,30]. It can also act as a membrane-inserting viral release promoting viroporin through interaction with adaptor protein complex 3 [31,32]. Moreover, mutants deficient in agnoprotein are severely impaired in viral DNA replication [28,33]. Less is known about BKPyV agnoprotein, but because of its high similarity with JCPyV (80% amino acid identity) and SV40 (63% amino acid identity) it is likely that these proteins exert similar functions. Indeed, cells transfected with BKPyV genomes deficient in the agnogene produce none or significantly less infectious particles compared to cells transfected with wild-type genomes. Furthermore, BKPyV agnoprotein can repress the activity of the late promoter [34,35]. Our group also demonstrated that BKPyV agnoprotein may interfere with secretion through its interaction with α-soluble N-ethylmaleimide-sensitive fusion attachment protein, a protein involved in disassembly of vesicles [36]. Interestingly, conditioned medium obtained from rat CG4-OI oligodendrocytes constitutively expressing JCPyV agnoprotein contain reduced amounts of several chemokines [37]. It is however, not known whether JCPyV agnoprotein interferes with the expression or the secretion of these chemokines.

Proliferating cell nuclear antigen (PCNA) is an essential component of the eukaryotic chromosomal DNA replication machinery that together with DNA polymerase (pol) δ ensure continuous DNA synthesis after DNA polymerase α/primase synthesized the RNA/DNA primer [38-41]. Topologically, PCNA exists as a hollow toroidal homotrimeric protein composed of individual 29 kDa subunits. One subunit consists of two lobes of β-sheets and α-helices connected by the “interstrand connecting loops” [42,43]. The α-helices form a positively charged inner ring that contacts the DNA, whereas the β-sheets form an outer rim with an overall negative charge distribution that provides an interaction platform for other proteins. More than 100 different PCNA interaction partners have been described so far [38,44,45]. These interaction partners participate in a variety of cellular processes such as DNA replication and repair, DNA methylation, histone acetylation, sumoylation, cell cycle control, and cell survival [38,46].

In the presented work we identified PCNA as a bona fide BKPyV agnoprotein interaction partner and provide evidence that agnoprotein inhibits PCNA-dependent pols δ DNA synthesis *in vitro*. The activities of DNA repair pols β and λ were unaffected by the presence of agnoprotein. Moreover, overexpression of agnoprotein reduced DNA synthesis in cell culture. Overexpression of PCNA counteracted the inhibitory effect of agnoprotein on DNA synthesis. Although we did not investigate the effect of agnoprotein on viral DNA replication, we suggest a similar role since viral DNA synthesis depends on PCNA and polδ. Turning off viral replication allows viral genomes to be packed into infectious viral particles.

## Results

### BKV agnoprotein and PCNA interact in vitro and in vivo

**Figure 1:**
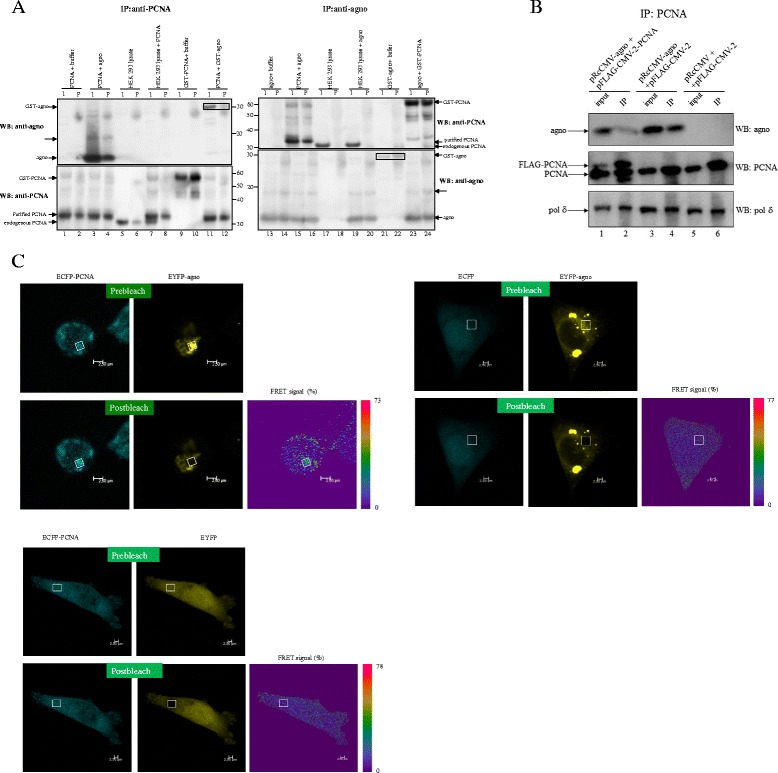
**BKPyV agnoprotein and PCNA interact** ***in vitro*** **and** ***in vivo.*** **(A)** *In vitro* pull down experiment shows direct interaction between PCNA and agnoprotein. Purified agnoprotein, PCNA or GST-PCNA fusion protein were incubated alone or together in PBS (buffer) or with lysate of HEK293 cells (cell lysate) at 4°C for 1 h. Complexes were immunoprecipitated with PCNA antibodies (IP:anti-PCNA; lanes 1–12) or with antibodies against agnoprotein (IP:anti-agno; lanes 13–24). Samples were run on gel and western blot was performed with antibodies against PCNA (WB:anti-PCNA) or against agnoprotein (WB: anti-agno) as indicated. I = input, P = precipitate. The position of GST-agno, agnoprotein, GST-PCNA, purified PCNA and endogenous PCNA are indicated by arrows. The arrow with dashed line probably represents agnoprotein dimers (see also Figure 2B). **(B)** Co-immunoprecipitation of agnoprotein and PCNA. HEK293 cells were transfected with following plasmids: lanes 1 and 2: pRcCMV-agno plus pFLAG-CMV-2-PCNA, lanes 3 and 4: pRcCMV-agno plus pFLAG-CMV-2, lanes 5 and 6: pRcCMV plus pFLAG-CMV-2. Lanes 1, 3, and 5: input; lanes 2, 4, and 6: immunoprecipitates (IP). Protein complexes were precipitated with antibodies against PCNA and the presence of polδ, PCNA and agnoprotein was examined with antibodies against these proteins. **(C)** FRET measurements of the interaction between ECFP-PCNA (donor) and EYFP-agnoprotein (acceptor) fusion proteins in A375 cells by acceptor photo bleaching. FRET efficiency (FRET signal %) is calculated by fluorescence before (prebleach) and after bleaching (postbleach) and shown by the colour code bar. Control experiments with ECFP plus EYFP-agno and ECFP-PCNA plus EYFP were included.

To elaborate on the function of BKPyV agnoprotein, we applied a yeast two hybrid screen using agnoprotein as bait against human kidney and thymus libraries and identified PCNA as a possible interacting partner (results not shown). To assure that this interaction takes place *in vitro* and is direct, we first performed pull down experiments with purified agnoprotein and PCNA (Figure 1A). Purified PCNA was mixed with buffer (lanes 1 and 2), agnoprotein (lanes 3 and 4), lysate from non-transfected HEK293 cells (lanes 7 and 8), or GST-agno (lanes 11 and 12). GST-PCNA was also added to buffer (lanes 9 and 10). Complexes were subsequently immunoprecipitated (IP) with antibodies directed against PCNA. The presence of proteins in the input (I) and the immunoprecipitates (P) was monitored by using antibodies against agnoprotein (WB: anti-agno; top panel left figure) and PCNA (WB: anti-PCNA; bottom panel left figure). Similarly, purified agnoprotein was incubated with buffer (lanes 13 and 14), PCNA (lanes 15 and 16) and cell lysate (lanes 19 and 20), or with GST-PCNA (lanes 23 and 24). GST-agno with buffer and lysate of non-transfected HEK293 cells alone were used as controls (lanes 17 and 18, and lanes 21 and 22). The reciprocal immunoprecipitation with anti-agno antibodies was followed by western blot with anti-PCNA antibodies (top panel, right figure) and anti-agno antibodies (bottom panel, right figure). Our results show a direct in vitro interaction between (GST)-PCNA and (GST)-agnoprotein (lanes 4, 12, 16 and 24). A band corresponding to ~18 kDa was detected with anti-agno antibodies in samples containing purified agnoprotein (lanes 3, 4, 13–16, 19, 20, 23 and 24). This corresponds probably to agnoprotein dimers (see further). It should be noticed that purified PCNA has some additional amino acids due to the cloning and this is reflected that the protein migrates a bit slower than endogenous PCNA (e.g. compare lanes 1–4 with 5 and 6, and lanes 15 and 16 with 17 and 19).

To access the existence of agnoprotein:PCNA in cells, HEK293 cells were transfected with expression plasmids for agnoprotein and either empty vector or a plasmid encoding FLAG-tagged PCNA. Complexes were subsequently immunoprecipitated (IP) with antibodies directed against PCNA. Agnoprotein could be detected in endogenous PCNA/FLAG-PCNA and endogenous PCNA immunoprecipitates derived from cells transfected with agnoprotein expression plasmid (Figure 1B, lanes 2 and 4, respectively), but not in cells not expressing agnoprotein (lane 6). Since PCNA can interact with pol δ, we tested whether immunoprecipitation of PCNA resulted in co-immunoprecipitation of pol δ. Indeed, pol δ was present using anti-PCNA antibodies in the co-immunoprecipitation study (bottom panel Figure 1B). These results suggested that PCNA and agnoprotein interact *in vivo* and that the binding of agnoprotein to PCNA did not disrupt the interaction between the latter protein and pol δ. We also tried the reciprocal experiment in which immunoprecipitation was done with anti-agnoprotein followed by western blot with anti-PCNA antibodies. However, this did not work in our hands (see also lane 20 in Figure 1A). Previous studies and our unpublished results demonstrate that agnoantibodies and PCNA antibodies do not cross-react with GST or other proteins in different cell lysates. Control experiments with nonspecific antibodies confirm the specificity of the PCNA:agnoprotein interaction (results not shown and [47]).

Förster or fluorescent resonance energy transfer (FRET) relies on distance-dependent energy transfer from a donor molecule to an acceptor. Because FRET can measure molecular proximity of less than 10 nm, this method is often used to examine protein-protein interaction in cells. The Förster radius *R*_*0*_ is defined as the distance at which 50% of the energy is transferred and the *R*_0_ value for the donor-acceptor CFP/YFP FRET pair is 4.92 nm [48,49]. To pursue the physical interaction between agnoprotein and PCNA, we performed FRET by acceptor photobleaching. A375 cells were chosen because they give very high transfection efficiency and do not easily detach from the wells of the chamber slides. We observed a FRET efficiency of 25-30% (Figure 1C), strongly indicating a physical interaction between agnoprotein and PCNA. For comparison, a positive control existing of the mitogen-activated protein kinase MK5 and heat shock protein 40 gave a FRET-efficiency of approximately 15% (results not shown). No interaction was observed when using expression plasmids for ECFP and EYFP-agno or ECFP-PCNA and EYFP. Taken together, these findings support that BKPyV agnoprotein and PCNA can also interact *in vivo*.

### Residues throughout the entire agnoprotein are required for efficient interaction with PCNA

**Figure 2:**
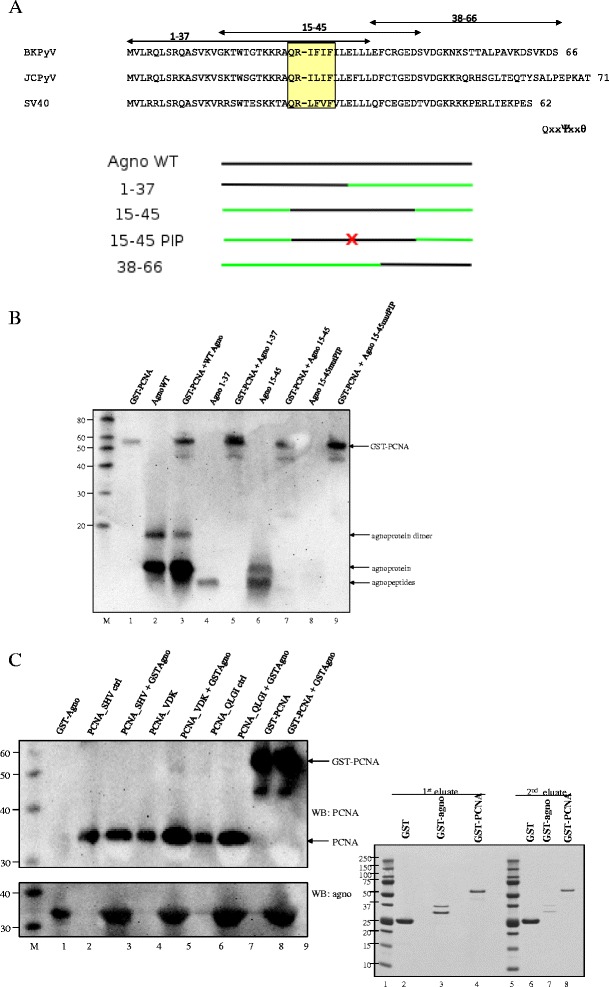
**Mapping of the agnoprotein and PCNA regions required for interaction. (A)** Amino acid sequences of the agnoproteins of BKPyV, JCPyV, and SV40 (top part) and schematic representation of BKPyV wild-type agnoprotein (agno WT) and truncated peptides (bottom part). The conserved “PIP box like sequence” is indicated. The regions encompassing peptides 1–37, 15–45, and 8–66 are indicated by arrows. The number at the end of the amino acid sequence refers to the number of residues in the protein. The name of the peptides corresponds to the residue numbers. PIP signifies the PCNA Interaction Protein box and the mutation QRIFIF into ARAFIA is symbolized by an X. **(B)** GST-pull down of purified GST-PCNA fusion protein and purified wild-type agnoprotein or agnopeptides. GST-PCNA (lanes 1, 3, 5, 7, 9) was mixed with full-length agnoprotein (lane 3) or agnoprotein peptide fragments comprising residues 1–37 (lane 5), residues 15–45 (lane 7) or residues 15–45 with mutated PIP motif (lane 9), respectively. The presence of full-length agnoprotein or peptides and PCNA was monitored by immunoblotting using antibodies against agnoprotein and PCNA simultaneously. The band representing agnoprotein dimer (lanes 2 and 3) is indicated. The additional weaker bands in lanes 3, 5, 7 and 9 probably represent degradation products. **(C)** GST-pull down experiments using purified GST-agnoprotein. Left panel: GST-agnoprotein was incubated with different mutants of PCNA (lanes 3, 5, 7) or GST-PCNA (lane 9) and complexes were pulled down. The presence of agnoprotein and PCNA was investigated by immunoblotting using antibodies against agnoprotein and PCNA, respectively. M = molecular mass marker in kDa. Right panel: Coomassie blue staining of purified GST (lanes 2 and 6), GST-agno (lanes 3 and 7) and GST-PCNA (lanes 4 and 8). Lanes 1 and 5: Precision Plus Protein Dual Color Standards (BioRad) marker.

Several PCNA interaction partners contain a PCNA Interacting Protein box (PIP motif) with consensus sequence QXXΨXXθ (Ψ = I/M/L, θ = F/Y, X = any amino acid; [38,50]). A motif with remote similarity is conserved in the agnoprotein of BKPyV (QRIFIF), JCPyV (QRILIF) and SV40 (QRLFVF) (Figure 2A). To examine whether this PIP motif is involved in the interaction with PCNA, we generated an agnoprotein mutant in which the PIP-like QRIFIF motif was substituted by ARAFIA (agno_mutPIP_) and monitored its ability to interact with PCNA. Unfortunately, this mutant was poorly expressed (Additional file 1: Figure S1). Therefore, we tried another approach, namely to map the amino acid residues of agnoprotein required to bind PCNA using pull down experiments with peptide fragments of agnoprotein. Purified agnoprotein or peptide fragments spanning amino acids 1–37, 15–45 or 38–66 and PCNA were incubated. We also used a peptide in which the PIP-like motif QRIFIF was replaced by ARAFIA (15-45mutPIP). Only full-length agnoprotein bound PCNA under the experimental conditions (Figure 2B, lane 3), whereas agnopeptides 1–37, 15–45 and 38–66 failed to do so (Figure 2B, lanes 5 and 7 and results not shown). This may indicate that several domains on agnoprotein are crucial for its interaction with PCNA. Similar to peptide 15–45, GST pull down did not reveal an interaction between GST-PCNA and peptide 15-45mutPIP. However, as agnoprotein antibodies did not recognize this peptide (Figure 2B, lane 8), we cannot completely exclude that peptide 15-45mutPIP was present in the GST-PCNA pull down complex.

Anti-agnoprotein antibodies detected a double band for agnopeptide 15–45 (lane 6), but not for agnopeptide 1–37 (lane 4). JCPyV agnoprotein was shown to form dimers that withstand strong denaturating conditions such as prolonged heating at 95°C, 8 M urea and 20% SDS. Dimerization of JCPyV agnoprotein requires residues 17–42 [26].

The dimerization domain is highly conserved in BKPyV agnoprotein (Figure 2A), and in fact BKPyV agnoprotein was also shown to form dimers [26]. Therefore, the upper band with agnopeptide 15–45 (lane 6) most likely represents dimers of 62 (2×31) amino acids compared to 66 amino acids of full-length BKPyV agnoprotein. Agnopeptide 1–37 (lane 4) probably lacks the residues crucial for dimerization and therefore no double band is observed (Figure 2B, lane 4). The weaker band of approximately 45 kD in lanes 3, 5, 7 and 9 most probably derives from degradation product ([51]; see also lanes 8 and 9 in left panel Figure 2C and lanes 4 and 8 in right panel Figure 2C).

We performed surface plasmon resonance experiments using Biacore T100. Again we found that full-length agnoprotein bound PCNA (Additional file 2: Figure S2), but the peptides either did not bind or displayed unspecific binding to the chip (results not shown). In conclusion, our data suggest that the whole agnoprotein rather than a specific domain of agnoprotein may be necessary for binding PCNA.

Next, we wanted to map the region of PCNA that is involved in the interaction with agnoprotein. The following PCNA mutations to Ala were used [52]: PCNA_*SHV43AAA*_, PCNA_*VDK188*_, PCNA_*QLGI125*_, and PCNA_*LAPK251AAAA*_. Mutation of residues SHV43 within the βC1-βD1 loop results in increased pol δ processivity, while mutation of QLGI125 reduces PCNA-mediated stimulation of DNA polymerase δ activity [38]. VDK188 in the loop between βD2 and βE2 is part of the most eye-catching feature in the PCNA structure and the residues are conserved in plant and vertebrate PCNA. Likewise, the LAPK251 motif is conserved in all known PCNA sequences [52,53]. The PCNA_*LAPK25AAAA*_ mutant could not be used since this one is unstable [our observation and 52]. *In vitro* pull down assays demonstrated that none of these mutations affected the binding of agnoprotein (Figure 2C). These results demonstrate that amino acid residues of PCNA which are involved in the interaction with other proteins are not required for agnoprotein binding.

### Agnoprotein affects PCNA-dependent DNA replication in vitro

**Figure 3:**
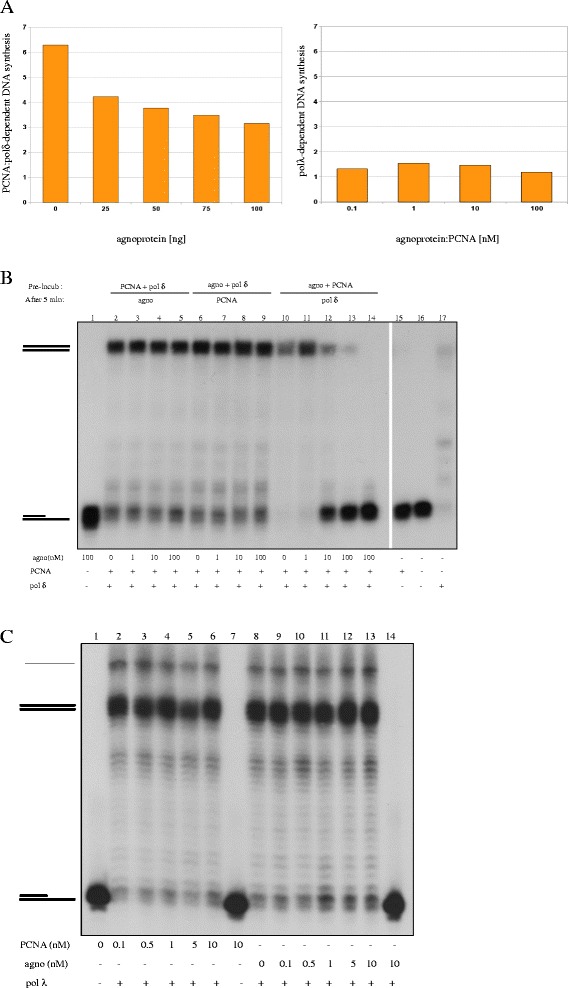
**Agnoprotein inhibits** ***in vitro*** **DNA replication mediated by PCNA:DNApolymerase δ. (A)** Left panel: *In vitro* DNA synthesis by PCNA plus pol δ was monitored in the presence of increasing amounts of agnoprotein. DNA synthesis in the presence of pol δ alone was arbitrary set as 1 and the increase by adding PCNA is shown as fold PCNA stimulation. Right panel: Pol λ-mediated DNA synthesis was monitored in the presence of variable nM ratios of agnoprotein:PCNA. Incorporation of radioactivity was measured by scintillation counting. **(B)** DNA synthesis was assayed by monitoring elongation of a partially double-stranded 39:72 oligonucleotide dimer (=template DNA). Lanes 1 and 16: ^32^P-labelled template DNA; lanes 2–5: PCNA and Pol δ were allowed to interact before template DNA and increasing amounts of agnoprotein were added; lanes 6–9: agnoprotein and pol δ were pre-incubated before supplementing PCNA and template DNA; lanes 10–14: agnoprotein and PCNA were mixed prior to the addition of pol δ and template DNA; lane 15: PCNA and template DNA; lane 17: template DNA and pol δ. The upper symbol (=) represents the elongated DNA template, while the lower symbol (− ) is the template. **(C)** Pol λ-mediated DNA synthesis in the presence of PCNA or agnoprotein. The upper band is ssDNA, the middle band is dsDNA, while the lower band represents incomplete dsDNA. Lanes 2–6: increasing amounts of PCNA were added; lanes 9–14: increasing amounts of agnoprotein were added; lane 1: template DNA; lane 8: DNA was incubated with only pol λ. DNA synthesis was visualized by autoradiography as described in 3B. The upper symbol (=) represents the elongated DNA template, while the lower symbol (− ) is the template.

Since PCNA is involved in DNA replication, a putative role for the agnoprotein may lay in stimulating or turning off viral DNA replication late in the viral life cycle by targeting PCNA. Because agnoprotein expression occurs at a later stage of the infection cycle, it is more probable to assume that agnoprotein suppresses viral DNA replication by perturbing PCNA’s function and allowing assembly of virions. We first examined whether agnoprotein could interfere with PCNA-mediated *in vitro* DNA replication. Because PCNA acts as a sliding platform for pol δ in DNA replication, we measured the effect of agnoprotein on PCNA:pol δ-mediated DNA synthesis. Decreased DNA synthesis activity was registered in the presence of increasing agnoprotein concentrations. PCNA:pol δ-mediated DNA replication was reduced by approximately 50% when 100 ng agnoprotein were added (Figure 3A left panel). In contrary, PCNA alone (results not shown) or together with increasing concentrations of agnoprotein were unable to affect pol λ-mediated DNA synthesis (Figure 3A, right panel). To assure that inhibition of DNA synthesis by agnoprotein occurred through interaction with PCNA and is not a direct effect on pol δ, we pre-incubated different combinations of two proteins for 5 min (either PCNA plus pol δ, pol δ plus agnoprotein, or PCNA plus agnoprotein) thus allowing them to interact, followed by the addition of the third protein and the primer/template DNA. The products of DNA synthesis were separated under denaturing conditions on an acrylamide gel. No inhibitory effect was observed in case of pre-incubation of PCNA and pol δ and pursued with agnoprotein (Figure 3B, lanes 2–5) or pre-incubation of agnoprotein and pol δ, followed by the addition of PCNA (Figure 3B, lanes 6–9). However, allowing PCNA and agnoprotein to interact prior to the addition of pol δ, suppressed DNA synthesis (Figure 3B, lanes 10–14). PCNA alone did not support DNA replication (Figure 3B, lane 15), while pol δ alone is able to synthesize DNA, although less processive when compared to the reaction in which PCNA is present as well (Figure 3B, compare lane 2 and lane 17). The reduced signal in lanes 10 and 11 can probably ascribed to some variation in the template input.

To assure that agnoprotein does not act as an unspecific inhibitor of the pol activity, a dose–response experiment with increasing concentrations of agnoprotein in the presence of constant amounts of pol λ was set up. Pol λ alone, but neither PCNA nor agnoprotein, was able to trigger DNA elongation on an incomplete double stranded oligonucleotide (compare lanes 7, 8, and 14 in Figure 3C). As pol λ is a PCNA-independent pol, no changes in DNA synthesis were visible when increasing amounts of PCNA were added to pol λ (Figure 3C, lanes 2–6). Likewise, agnoprotein had no effect on *in vitro* pol λ-mediated DNA elongation (Figure 3C, lanes 9–13). Accordingly, agnoprotein did not interfere with pol β-mediated DNA synthesis (results not shown). In conclusion, our results suggested that the agnoprotein specifically inhibits pol δ-mediated DNA synthesis *in vitro* through targeting PCNA, but does not affect PCNA-independent synthesis mediated by the repair pol λ.

### Agnoprotein affects DNA replication in vivo

**Figure 4:**
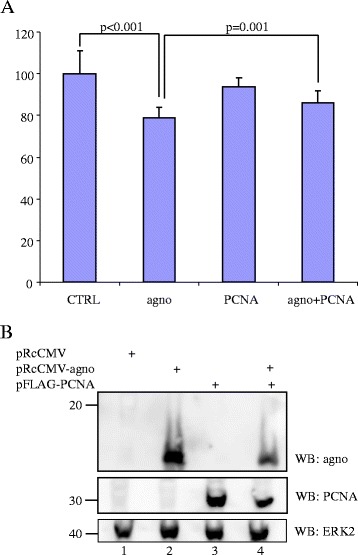
**Agnoprotein inhibits cell proliferation.** A375 cells were transfected with empty expression plasmid pRcCMV (CTRL), expression plasmid for agnoprotein (Agno), expression plasmid for PCNA, or expression plasmids for agnoprotein and PCNA, respectively. **(A)** Cell proliferation was measured by the MTT method 48 hours after transfection. The value obtained for pRcCMV-transfected cells was arbitrary set as 100% and the values for the other cells were related to this. The values are the average of six independent parallels measured in triplicate. The standard deviation is shown and a student’s t-test was performed. Similar results were obtained in a separate experiment. **(B)** Ectopically expressed agnoprotein and FLAG-tagged PCNA were detected by western blot on lysates of transfected cells using anti-agnoprotein and anti-FLAG-specific antibodies. Lane 1: lysates of cells transfected with empty expression plasmid pRcCMV; lane 2: lysates of cells transfected with pRcCMV-agnoprotein expression plasmid; lane 3: lysates of cells transfected with an expression plasmid encoding FLAG-tagged PCNA; lane 4 lysates of cells co-transfected with expression plasmids for agnoprotein and FLAG-tagged PCNA. To assure equal loading, expression levels of ERK2 in each lysate were determined (bottom panel).

Next, we wanted to access an effect of agnoprotein on PCNA-mediated DNA replication *in vivo*. A375 cells were transfected with expression plasmids for agnoprotein or/and PCNA. Control cells were transfected with empty expression plasmid. A375 were chosen because they have very high transfection efficiency and in contrast to HEK293 cells they do not easily detach. Forty-eight hours after transfection, cell proliferation was measured by the MTT assay (Figure 4). Ectopic expression of agnoprotein inhibited cell proliferation by 21% (p < 0.001). Overexpression of PCNA had no significant effect on cell proliferation (94% proliferation compared to control; p = 0.04). Co-expression of agnoprotein and PCNA partially restored agnoprotein-mediated inhibition of cell proliferation to 86% compared to control cells (p = 0.001). These results suggest that agnoprotein-induced repression of cell proliferation involves PCNA.

## Discussion

The role of PyV agnoprotein remains puzzling because the gene encoding this protein is not present in the genomes of all members of the *Polyomaviridae*, while all other viral genes are well-conserved. To unveil the biological importance of BKPyV agnoprotein, we set out to identify cellular interaction partners by a yeast two-hybrid screen. PCNA was found to bind directly to agnoprotein by GST-pull down assays. Co-immunoprecipitation and FRET studies confirm *in vivo* interaction between PCNA and agnoprotein. Agnoprotein:PCNA complexes could be co-immunoprecipitated using anti-PCNA antibodies, but the reciprocal precipitation did not work in our hands even though we tried different experimental conditions. We reason that since agnoprotein is small (66 amino acids), its interaction with PCNA may prevent anti-agnoprotein antibodies to bind due to steric hindrance. We therefore tried immunoprecipitation of GAL4-tagged agnoprotein (147 amino acid tag) with anti-GAL4 antibodies, but were also unsuccessful. The reason is unknown, but the GAL4 moiety may change the conformation of agnoprotein, making it impossible to interact with PCNA. Studies with shorter tags (e.g. HA or myc) may circumvent this problem.

### Regions required for agnoprotein:PCNA interaction

Many of the PCNA interacting proteins contain a PIP box with consensus sequence QxxI/M/LxxF/Y [38,50]. BKPyV agnoprotein contains a QRIFI motif with remote homology to the PIP box. Agnopeptide fragments spanning residues 1–37 and 15–45 were unable to bind PCNA, despite the presence of the putative PIP-like motif. Co-immunoprecipitation studies using extracts of cells transfected with a plasmid encoding full-length agnoprotein with PIP mutation were unsuccessful because this mutant was not expressed at detectable levels, probably due to instability of the protein. None of the agnopeptide fragments tested (peptides encompassing residues 1–37, 15–45 or 38–66) were able to interact with PCNA in our assay.

Several PCNA-interacting proteins have been isolated that lack an obvious PIP box. In fact, none of the 14 cytoplasmic proteins that bind PCNA contain the canonical PIP box [54]. Gilljam and colleagues identified another shared motif in PCNA-interacting proteins without a clear PIP box; the AlkB homologue 2 PCNA-interacting motif (APIM). The APIM motif consists of K/RFIVK/R flanked on both sides by a K and/or R. This motif is common in proteins involved in DNA maintenance and cell cycle regulation after DNA damage [55]. BKPyV agnoprotein does not contain an APIM-like sequence. None of the mutations in the SHV, VDK, QLGL motifs of PCNA abrogated the interaction with agnoprotein. Although some of these motifs are implicated in the interaction with cellular proteins such as pols δ and ε, replication factor C, p21 and flap endonuclease 1 [52,53], they are dispensable for binding agnoprotein.

### PCNA, agnoprotein, and DNA polymerase δ: binary or ternary complexes?

Pull down with anti-PCNA antibodies of lysates derived from cells transfected with the agnoprotein expression plasmid or with empty vector revealed the presence of comparable pol δ levels in the immunoprecipitates. This may indicate that agnoprotein and pol δ bind PCNA in a non-exclusive manner to form a ternary complex. Indeed, if agnoprotein would compete with pol δ to bind PCNA, less pol δ would be expected in immunoprecipitates of cells expressing agnoprotein compared to non-agnoprotein expressing cells. Agnoprotein and pol δ may bind to different regions of PCNA or pol δ is the bridging molecule that connects agnoprotein and PCNA. *In vitro* GST-pull down studies, however, demonstrated a direct interaction between PCNA and agnoprotein. We have not investigated whether agnoprotein bound to PCNA also interacts with pol δ.

### Mode of action for agnoprotein-mediated inhibition of PCNA: DNA polymerase δ DNA replication

The precise mechanism by which agnoprotein suppresses PCNA:pol δ-mediated DNA synthesis remains unsolved. One obvious possibility is that agnoprotein disrupts the interaction between PCNA and pol δ. However, studies with lysates of cells overexpressing agnoprotein and PCNA, showed that protein complexes immunoprecipitated with PCNA antibodies contained all three proteins: PCNA, pol δ, and agnoprotein (Figure 1B). This indicates that agnoprotein does not disrupt the interaction between PCNA and pol δ. Another mechanism is that binding of agnoprotein to PCNA neutralizes the enzymatic activity of PCNA or prevents the PCNA:pol δ complex to bind to template DNA. A third mechanism to explain the inhibitory effect of agnoproteins is by targeting pol δ. Our *in vitro* DNA elongation studies do not indicate that agnoprotein alone hampers pol δ-driven DNA synthesis, but it is possible that agnoprotein after it is recruited by PCNA can inhibit pol δ activity, which is in accordance with our results. Finally, agnoprotein may negatively interfere with PCNA:pol δ-mediated replication by down-regulating the expression of PCNA. This seems unlikely because all our co-immunoprecipitation studies were done in cells overexpressing agnoprotein and we could vividly detect PCNA. Moreover, PCNA protein levels in cells overexpressing agnoprotein were similar to cells not expressing agnoprotein (compare lanes 1,3 and 5 in Figure 1B, and our unpublished results).

### Functional implication of the agnoprotein-PCNA interaction

Replication of the PyV genome depends mostly on host cell proteins. The only viral protein absolutely necessary for viral replication is large T-antigen [1]. Recent studies have shown that agnoprotein-deficient polyomaviruses JC and SV40 display lower viral DNA replication, but also reduced large T-antigen expression levels compared to wild-type viruses [33,56]. A difference between cellular and viral DNA replication seems to be the involvement of different polymerases. The host cell requires pol ε for the replication of the leading strand, while pol δ synthesizes the lagging strand [39,41]. However, pol δ is also capable of performing leading strand synthesis [39,41]. This is illustrated during SV40 infection, when DNA replication only relies on pol δ [57,58]. Pol δ is a PCNA-dependent pol [38]. As the viral replication cycle proceeds, a switch from producing viral genomes to synthesizing viral capsid proteins and assembling virions is required. Agnoprotein could play a regulatory role by turning of viral DNA synthesis through inhibiting PCNA:pol δ-mediated DNA replication. Because cellular DNA replication also depends on pol δ, agnoprotein will also suppress this process. Indeed, we found that expression of agnoprotein inhibited cell proliferation and this effect was counteracted by co-expressing PCNA.

Viruses can prevent apoptosis, which allow them to replicate. They can also induce apoptosis to enable viral shedding from the infected cells [59]. By leaving within apoptotic bodies, viruses may spread to surrounding cells and macrophages without provoking a strong immune/inflammatory response [60-62]. PCNA has been shown to prevent apoptosis in neutrophils [63]. As agnoprotein is expressed late during the viral infection cycle, it is plausible that agnoprotein triggers apoptosis of the infected cell in order to disseminate newly produced virions. Indeed JCPyV agnoprotein has been shown to promote apoptosis, but the implication of PCNA was not investigated [37,64]. Neutrophils undergoing apoptosis witness a degradation of PCNA by the proteasome, while overexpression of PCNA rescues apoptosis of neutrophils. This anti-apoptotic property of PCNA depends on cytoplasmic location of PCNA [63]. Because we observed that BKPyV agnoprotein did not reduce the expression levels of PCNA (see above), it might exert its apoptotic effect by sequestering cytoplasmic PCNA. PCNA:DNA pol δ is also involved in the base excision repair pathway [46]. The interaction of agnoprotein with PCNA may therefore perturb PCNA:DNA pol δ − dependent DNA repair, resulting in an accumulation of mutations. Agnoprotein may in this way contribute to transformation of BKPyV-infected cells.

## Conclusions

Our study identifies PCNA as a novel BKPyV agnoprotein interaction partner and demonstrates that agnoprotein impedes with the auxiliary function of PCNA in pol δ-mediated DNA synthesis. Given the fact that agnoprotein is expressed late during the viral cycle, it would seem plausible that such an interaction serves to terminate viral DNA replication so that virus particles can be assembled. At the same time, agnoprotein may promote apoptosis in a PCNA-dependent manner and enhance the release of virions. Both these putative roles of agnoprotein need further investigations. Agnoprotein does not only facilitate viral dissemination, it also possesses oncogenic potentials and has been detected in human tumours. Its role in carcinogenesis, however, remains to be proven [65,66]. One of the oncogenic properties could rely on agnoprotein’s ability to increase the error rate of DNA replication and repair by targeting PCNA. Agnoprotein may therefore be an attractive target in malignant and non-malignant pathologies.

## Methods

### Materials

PCNA (C-10) and pol δ (p125) antibodies were from Santa Cruz Biotechnology (Santa Cruz, CA, USA), while ERK2 was purchased from Cell Signalling Technologies, Inc (Danvers, MA, USA). Antibody against BKPyV agnoprotein has been described previously [67]. Alkaline phosphatase-conjugated and horse radish peroxidase-conjugated secondary antibodies were purchased from Dako (Denmark). Thrombin protease was from Amersham Biosciences (GE Healthcare Bio-Sciences AB, Uppsala, Sweden). The agnopeptides residues 1–37 (MVLRQLSRQASVKVGKTWTGTKKRAQRIFIFILELLL), residues 38–66 (EFCRGEDSVDGKNKSTTALPAVKDSVKDS), residues 15–45 (GKTWTGTKKRAQRIFIFILELLLEFCRGED), and residues 15-45mutPIP (GKTWTGTKKRA**A**R**A**FI**A**ILELLLEFCRGED) were synthesized by Gene Cust (Luxembourg) and were dissolved in H_2_0. Primers for site-directed mutagenesis (the complementary primer is not shown) *PIP box* (5’-A AAA AGA GCT GCG AGG GCT TTT ATT GCT ATT TTA GAG C-3’), *K164R* (5’ GTA ATT TCC TGT GCA AGA GAC GGA GTG 3’), PCNA_SHV43 (5’- CAT GGA CTC GGC CGC CGC CTC TTT GGT-3’), PCNA_VDK188 (5’- ACA AGT AAT GCC GCT GCA GAG GAG GAA-3’), and PCNA_QLGI125 (5’- TAG ATG TTG AAG CCG CTG CAG CTC CAG AAC A-3’) were from Sigma Aldrich (St. Louis, MO, USA). The sequence of the 72-mer is: ATGTTGGTTCTCGTATGCTGCCGGTCACGGCTTAAGTGT**G**GCGGCCGCGGGTTGGAGGGCTTATAGATTATG, and the sequence of the 39-mer is complementary to the underlined region in the 72-mer [68].

### Plasmids

The empty expression vector pRcCMV was purchased from Invitrogen (Invitrogen Life Technologies, Grand Island, NY, USA). The plasmid pRcCMV-agno which encodes BKPyV agnoprotein has been previously described [7]. Plasmid pFLAG-CMV-2 was obtained from Sigma Aldrich, while pFLAG-CMV-2 PCNA expression vector was a kind gift of Hong-Gang Wang [69]. pGST-agno has been described previously [34].

### Cells

HEK 293 cells (ECACC cat. No. 85120602) were purchased from the European Collection of Cell Culture (ECACC) and were maintained in DMEM with 10% foetal bovine serum (FBS; Sigma Aldrich) in the presence of 100 μg/ml streptomycin and 100 units/ml penicillin. The human melanoma cell line A375 was obtained from American Type Culture Collection (ATCC cat. no. CRL-1619) and kept in DMEM supplemented with 10% FBS, streptomycin (100 μg/ml), and penicillin (100 units/ml).

### Western blot

Samples were analysed by 10-20% SDS-PAGE Bis-Tris glycine ClearPage gels (Invitrogen Life Technologies) according to the manufacturer’s protocol and blotted onto a 0.45 μm PVDF membrane (Millipore, Billerica, MA, USA). Immunoblotting was performed by first blocking the membrane with PBS-T (PBS with 0.1% Tween-20; Sigma Aldrich) containing 10% (w/v) dried skimmed milk for 1 hour and probed with the appropriate primary antibody overnight at 4°C. After 3 washes, the membrane was incubated with the appropriate secondary antibody for 1 hour at room temperature. After 4 washes, antigen-antibody complexes were visualized using CDP Star (Tropix, Bedford, MA, USA) substrate and Lumi-Imager F1 from Roche (Basel, Switzerland). Magic-Mark™ Western standard from Invitrogen Life Technologies was used to estimate the molecular mass of the detected proteins.

### Immunoprecipitation

HEK293 cell extracts were harvested and lysed in buffer containing PBT (PBS + 0.1% Triton X100) and Complete Protease Inhibitor Cocktail (Roche Diagnostics Norway). Lysates were cleared by centrifugation at 4°C for 10 minutes at 15,000 g and incubated with the appropriate antibody for 1 hour at 4°C, before addition of 60 μl slurry (i.e. 30 μl protein G-agarose (GE Healthcare) equilibrated with 30 μl lysis buffer) and incubated for an additional hour. The immunoprecipitates were then washed three times in lysis buffer and twice in 20 mM Hepes pH 8.0. Twenty μl 2xLDS sample buffer was added to the beads before denaturation at 70°C for 10 minutes. The immunoprecipitates were analysed on western blots.

### Protein purification

GST-agnoprotein and mutant proteins were purified from *Escherichia coli* BL21 bacteria according to the protocol of the manufacturer. Briefly, GST-agarose beads were washed three times with PBT (PBS + 0.1% TritonX100). After centrifugation, bacteria were lysed in PBT containing protease inhibitor cocktail and the lysate was cleared by centrifugation. The supernatant was transferred to 15 ml tubes and 50% beads were added for 1 h incubation at 4°C. Then the beads were collected and washed twice with PBT and twice with PBS. GST cleavage was performed with thrombin for 2 h at RT. Alternatively, glutathione was added and two elution steps were used to assure that all GST-coupled protein was recovered. GST and GST fusion proteins were dissolved in PBS. Protein yield was checked on SDS-PAGE gels with Coomassie staining. Pols β, δ, λ and PCNA recombinant proteins were purified as previously described [68,70].

### Yeast two hybrid screen

Yeast-two hybrid screen was performed by PanBionet (Pohang, South Korea) with BKPyV agnoprotein as bait against human kidney and thymus libraries. Transformants were spread on selection media (SD-LWU) where yeasts with bait and prey interacting together could theoretically grow. The yeast colonies on uracil deficient media were analysed for other reporter expression (lacZ and ADE2) under the control of a different GAL4-dependent promoter. Through the screening of the two libraries, 53 Ura + colonies were obtained, 28 from the kidney library and 25 from the thymus library. Only 12 of colonies showed expression of other reporter genes *ADE2* and *lacZ* (3 from the kidney and 9 from the thymus library). Since only a small number of candidates were obtained, prey plasmids were isolated from all Ura + candidates from both libraries. The prey DNAs were amplified by PCR from the purified DNAs. In order to reconfirm the interaction, the preys were reintroduced into yeast with agnoprotein bait or with empty bait plasmid containing only GAL4-DNA binding domain lacking agnoprotein. Most of the prey plasmids tested showed self-activator activities. Some candidates which did not reproduce the reporter expression were retested with preys isolated by conventional *E. coli* transformation with yeast purified DNAs.This approach resulted in 6 putative interaction partners for BKPyV agnoprotein and one of them was PCNA.

### DNA replication assays

PolyA-oligodT served as a template for DNA replication assays presented in Figure 3A, while in Figures 3B-C labelled 39/72-mer primer template was used. Annealing of the polydA-oligodT (0.5 mg/ml) was done using a 10:1 polydA/oligodT ratio with Tris–HCl pH 8 for 5 min at 65°C in a heat block followed by addition of 20 mM KCl and left to cool down slowly. A 39-mer primer was labelled by incubating T4 Polynucleotide kinase buffer, Polynucleotide kinase enzyme (New England Bioloabs, Ipswich, MA, USA), ^32^P-γ ATP (3000 Ci/mmol; PerkinElmer Waltham, MA, USA) and 39-mers for 20 min at room temperature and 20 min at 37°C, heat-inactivated at 80°C for 10 min, centrifuged on MicroSpin columns (GE Healthcare Life Sciences) to remove the excess of unincorporated radioactive label. The radioactively labelled 39-mer was then annealed to a 72-mer template in annealing buffer, heated to 95°C for 10 min, and subsequently left to cool down overnight. The pol δ assays were performed in presence of 50 mM BisTris pH6.5, 1 mM DTT, 0.25 mg/ml BSA, 5 mM MgCl2 and 2 nM labelled 39/72-mer primer template (Figure 3) or 10 μM ^3^[H]dTTP (400 cpm/pmol), polydA/oligo dT (10 μg/ml) (Figure 3A, left panel) PCNA, pol δ and agnoprotein were added in quantities indicated in figures and figure legends. The reactions were incubated at 37°C for 30 minutes and stopped by addition of 10%TCA (Figure 3A, left panel) or stop buffer (2x stop buffer is 96% formamide (v/v), 20 mM EDTA, bromophenol blue and xylene cyanol) (Figure 3B). In the former case results were analysed by scintillation counting (V_*total*_=50 μl), in the latter case by sequencing gels (V_*total*_=25 μl). For the pol λ or β assays, λ buffer (10x λ buffer is 500 mM Tris–HCl pH7.5; 10 mM DTT, 2.5 mg/ml BSA), 0.8 mM MnCl_2_, , and 2 nM labelled 39/72-mer primer template (Figure 3C) or 20 μM polydA/oligo dT with 50 μM [^3^H]-dTTP (400 cpm/pmol) (Figure 3A, right panel) were prepared as a premix. Purified pols λ or β and agnoprotein were added to 25 μl reaction mixture containing polydA/oligo dT and [^3^H]-dTTP and incubated at 37°C for 30 min, followed by addition of 10% TCA and analysis by scintillation counting. Polymerase λ or β and agnoprotein were incubated with the premix containing labelled 39/72-mer primer template (Figure 3C) in a final volume of 10 μl. Reactions were incubated at 37°C for 15 min and stopped by addition of the stop buffer. Reaction products were analysed on a sequencing gel.

### MTT assay

A375 cells were seeded out at 5×10^4^ cells per well in a 24-well plate. The next day, cells were transfected by Lipofectamine 2000 (Invitrogen) with empty expression pRcCMV, or a plasmid encoding BKPyV agnoprotein, PCNA or both. The amount of DNA in each sample was kept constant by adding calf thymus DNA. Forty-eight hours after transfection, MTT (3-(4,5-dimethylthiazol-2-yl)-2,5-diphenyltetrazolium bromide; Sigma- Aldrich) was added to a final concentration of 0.5 mg/ml and the cells were incubated for 2 hours at 37°C. Then stop solution (isopropanol containing 0.04 N HCl) was added and the cells were incubated for 1 hour at room temperature. Thereafter, 3×100 μl of each well was transferred to new wells of a 96-well plate. Absorbance at 570 nm was measured in a Molecular Devices VERSA_max_ microplate reader (Molecular Devices LLC, Sunnyvale, CA, USA).

### Fluorescence resonance energy transfer (FRET) studies

The oligonucleotides 5’-GCAGTCGACATGTTCGAGGCGCGCCTGGTC-3’ and 5’- TATGCGGCCGCCTAAGATCCTTCTTCA-3’ were used to amplify the PCNA cDNA sequence from pFlag-CMV-PCNA. Then, the PCR fragment was cut with *Sal*I/*Not*I and ligated into the corresponding sites of pENTR1A (Invitrogen). The plasmid pGST-agno was digested with *Eco*RI/*Not*I, and the fragment ligated into the corresponding sites of pENTR3C (Invitrogen). The resulting pENTR-PCNA and pENTR-agno were further recombined with Gateway destination vectors pDest-ECFP and pDest-EYFP (a gift from Dr. T. Johansen), respectively, generating EYFP-agno and ECFP-PCNA. For FRET analysis, A375 cells were co-transfected with ECFP-PCNA and with EYFP-agno using TransIT-LT1 transfection reagent (Mirus Bio LLC, Madison, WI, USA). Then, 24 hrs after transfection, the cells were fixed with 4% formaldehyde. FRET analysis was carried out with Leica TCS SP5 confocal microscope with a × 63, 1.2 W objective. CFP and YFP were excited with separate laser channels of 458 nm and 514 nm, respectively. Emission fluorescence intensity data were obtained at 465–500 nm (CFP) and 525–600 nm (YFP). CFP and YFP emission signals were captured before and after 25% photo bleaching YFP. FRET is indicated as the relative increase in CFP emission following YFP photo bleaching. The imaging system was controlled by the Leica Application Suite Advanced Fluorescence (LAS AF) software (http://www.leicamicrosystems.com).

### BIAcore

The association between agnoprotein and PCNA were measured with a BIAcore T100 instrument (BIAcore, Uppsala, Sweden). Standard sensor chips, amine coupling kit containing N-hydroxysuccinimide, 1-ethyl-3-(3-dimethylaminopropyl) carbodiimide hydrochloride, running buffer 10xHBS-EP+, regeneration solution, immobilization buffers and 1 M ethanolamine hydrochloride, all purchased from Biacore (Biacore, Sweden), were used for the measurements. Analyses were performed with the Biacore T100 control software and Biacore T100 evaluation software 1.1 using the Langmuirmodel for 1:1 ligand interaction to determine the association (ka) and dissociation (kd) constants and calculation of the KD value.

## Additional files

Additional file 1: Figure S1. The agnoprotein mutant in the putative PIP motif is poorly expressed. HEK293 cells were transfected with expression plasmids for agnoprotein (agno; lane 2), agnoprotein in which the PIP-like motif was mutated (lane 3; agno_mutPIP_), or empty expression plasmid (empty vector, lane 4). Cell lysates were prepared and the expression of agnoprotein (top panel) or PCNA (bottom panel) was monitored by western blotting using anti-agnoprotein and anti-PCNA antibodies, respectively. Lane 1: protein size marker (in kD). Additional file 2: Figure S2. Biacore measurements to study *in vitro* interaction between recombinant PCNA and agnoprotein. PCNA (20–30 μg/ml) was immobilized on a CM5 chip. Binding properties of PCNA antibody (positive control), BSA (negative control) and WT agnoprotein (10 μg per injection) to PCNA were assessed. The sensorgram on the left depicts the results obtained with PCNA antibody (green) and BSA (blue), while the sensorgram on the right depicts the binding profile of WT agnoprotein (red). Responses are given in resonance units (RU) in function of time, where 1000RU corresponds to a change in 0.1 degree in resonance angle.

## Abbreviations

Pol: Polymerase
PyV: Polyomavirus
FRET: Fluorescence resonance energy transfer
PCNA: Proliferating cell nuclear antigen
