# Supplementary figures and images for: Agnoprotein of polyomavirus BK interacts with proliferating cell nuclear antigen and inhibits DNA replication
Source: Virol J. 2015 Feb 1;12:7. doi: 10.1186/s12985-014-0220-1 (PMC4318453; doi:10.1186/s12985-014-0220-1)

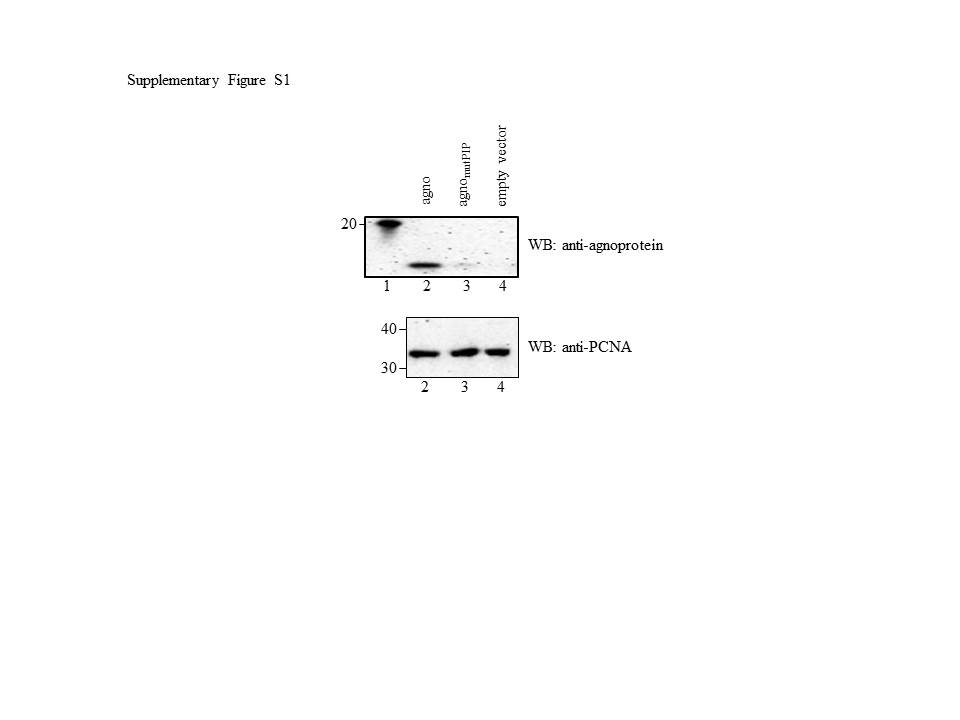

Supplement: Additional file 1: Figure S1. — The agnoprotein mutant in the putative PIP motif is poorly expressed. HEK293 cells were transfected with expression plasmids for agnoprotein (agno; lane 2), agnoprotein in which the PIP-like motif was mutated (lane 3; agnomutPIP), or empty expression plasmid (empty vector, lane 4). Cell lysates were prepared and the expression of agnoprotein (top panel) or PCNA (bottom panel) was monitored by western blotting using anti-agnoprotein and anti-PCNA antibodies, respectively. Lane 1: protein size marker (in kD). [file 12985_2014_220_MOESM1_ESM.jpeg]

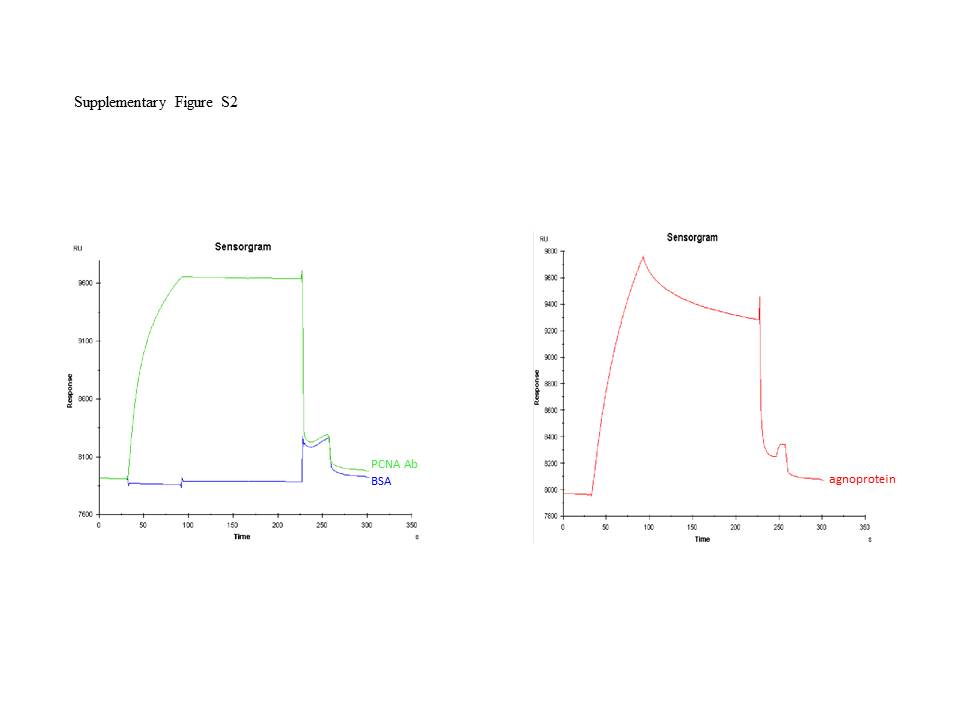

Supplement: Additional file 2: Figure S2. — Biacore measurements to study in vitro interaction between recombinant PCNA and agnoprotein. PCNA (20–30 μg/ml) was immobilized on a CM5 chip. Binding properties of PCNA antibody (positive control), BSA (negative control) and WT agnoprotein (10 μg per injection) to PCNA were assessed. The sensorgram on the left depicts the results obtained with PCNA antibody (green) and BSA (blue), while the sensorgram on the right depicts the binding profile of WT agnoprotein (red). Responses are given in resonance units (RU) in function of time, where 1000RU corresponds to a change in 0.1 degree in resonance angle. [file 12985_2014_220_MOESM2_ESM.jpeg]
